# Survival of HIV/AIDS patients treated under ART follow-up at the University hospital, northwest Ethiopia

**DOI:** 10.1186/s12199-021-00976-8

**Published:** 2021-04-30

**Authors:** Zinabu Teka, Kasim Mohammed, Gashu Workneh, Zemichael Gizaw

**Affiliations:** 1 Department of Statistics, College of Natural and Computational Sciences, University of Gondar, Gondar, Ethiopia; 2 Department of Environment and Occupational Health and Safety, Institute of Public Health College of Medicine and Health Sciences, University of Gondar, Gondar, Ethiopia

**Keywords:** HIV/AIDS, ART, Survival analysis, Cox proportional hazards model, Gondar University Teaching Hospital

## Abstract

**Introduction:**

The survival of HIV/AIDS patients on antiretroviral therapy (ART) is determined by a number of factors, including economic, demographic, behavioral, and institutional factors. Understanding the survival time and its trend is crucial to developing policies that will result in changes. The aim of this study was to compare the survival estimates of different subgroups and look into the predictors of HIV/AIDS patient survival.

**Methods:**

A retrospective cohort study of HIV/AIDS patients receiving ART at the University of Gondar teaching hospital was carried out. To compare the survival of various groups, a Kaplan-Meier survival analysis was performed. The Cox proportional hazards model was used to identify factors influencing HIV/AIDS patient survival rates.

**Results:**

In the current study, 5.91% of the 354 HIV/AIDS patients under ART follow-up were uncensored or died. Age (HR = 1.051) and lack of formal education (HR = 5.032) were associated with lower survival rate, whereas family size of one to two (HR = 0.167), three to four (HR = 0.120), no alcoholic consumption (HR = 0.294), no smoking and chat use (HR = 0.101), baseline weight (HR = 0.920), current weight (HR = 0.928), baseline CD4 cell count (HR = 0.990), baseline hemoglobin (HR = 0.800), and no TB diseases were associated with longer survival rate.

**Conclusions:**

Fewer deaths were reported in a study area due to high patient adherence, compared to previous similar studies. Age, educational status, family size, alcohol consumption, tobacco and chat usage, baseline and current weight, baseline CD4 cell count, baseline hemoglobin, and tuberculosis (TB) diseases were all significant predictors of survival of HIV/AIDS patients.

## Background

Acquired immune deficiency syndrome (AIDS) is the world’s most serious public health problem. It affects over 35 million people worldwide [1]. HIV infection has progressed from a fatal disease to a chronic illness, owing primarily to the development of ART [2]. Understanding the survival experience of AIDS patients, as well as the factors that influence survival, is critical for increasing understanding of the pathophysiology of the disease, clinical decision making, and planning health service interventions [3]. The main issue here is the progress and coverage of ART and other related medications at district hospitals.

According to previous research, approximately one million people in Ethiopia were infected with HIV in 2008 [4]**.** Survival trends following HIV infection in African populations prior to the introduction of ART were used as a baseline for assessing future success of intervention programs [5]. Despite the availability of a large body of research that confirms the facts about HIV/AIDS in Ethiopia, understanding levels about determinant factors associated with HIV patient survival time are negligible [6].

Moreover, even though ART treatment has proven to be clinically significant, a number of deaths occur that could be avoided with appropriate interventions on certain socioeconomic, demographic, behavioral, and institutional factors. This study was, therefore, conducted to compare the survival estimates of different subgroups and look into the predictors of HIV/AIDS patient survival.

## Methods

### Study design

A retrospective study design was used on people living with AIDS who were being treated with ART at the University of Gondar teaching hospital in Gondar, Ethiopia. The target population included all patients who received ART between September 2009 and March 2015. A total of 3397 HIV/AIDS patients (over the age of 14) were registered in the ART follow-up database.

### Sample size determination

The following sampling equation [7] was used to determine the sample size.

$$ n=\frac{\frac{Z^2p\left(1-p\right)}{d^2}}{1+\frac{1}{N}\left(\frac{Z^2p\left(1-p\right)}{d^2}-1\right)}=354 $$

where *Z* is the upper α/2 points of standard normal distribution with significance level, which is *Z* = 1.96. *d = 3.36% is* the degree of precision that mostly selected by the investigator. The term *p* represents the proportion of death among HIV/AIDS patients. The proportion of death *р* that was selected for this study was obtained from the previous comparable study done by [8] on data taken from Felege-Hiwot Referral Hospital which is 13.4%.

### Data collection techniques

Data were gathered by reviewing the medical records of 354 HIV patients. Three hundred and forty-four charts were chosen at random from a total of 3397 charts registered between September 2009 and March 2015. To collect information, a structured and pretested checklist was used.

### Estimation of the outcome variable

HIV/AIDS survival time (measured in months), the primary outcome variable of this study, was estimated from the date of HIV/AIDS diagnosis to the date of HIV/AIDS-related death or censoring. We measured patients’ survival experiences using demographic, clinical, and behavioral factors.

### Data analysis

The data was analyzed using SPSS version 20. Descriptive measures were used to investigate the proportion of patients who fell into different subgroups of each variable, as well as the mean survival time of patients in these subgroups. The Kaplan-Meier survival analysis was used to compare the estimated survival times of patients in different classifications. The Cox proportional hazards model was used to calculate the hazard of death for patients and to identify the factors associated with HIV/AIDS patients’ survival.

## Results

### Demographic information of the patients

There were 21 (5.93%) deaths among 354 HIV/AIDS patients, and 336 (94.07%) were censored. More than half of the patients, 211 (59.6%), were female. Almost all of the study subjects, 306 (86.4%), live in cities. One hundred sixty-two patients (45.8%) were married. A large number of patients, 320 (90.4%), were orthodox Christians. Of the total study participants, 201 (56.8%) had a family size of one to two people. One hundred sixty (45.2%) of the patients had a secondary education or higher (Table 1).

**Table 1:** Summary results for socio-demographic composition of HIV/AIDS patients treated under ART follow-up (University of Gondar teaching hospital, 2009–2015)

Variables	Frequency	Percent
Sex		
Female	211	59.6
Male	143	40.4
Residence		
Urban	306	86.4
Rural	48	13.6
Marital status		
Single	76	21.5
Married	162	45.8
Separated/divorced	90	25.4
Widowed	26	7.3
Religion		
Orthodox	320	90.4
Muslim	29	8.2
Other	5	1.4
Family size		
1–2	201	56.8
3–4	91	25.7
≥5	62	17.5
Educational status		
No formal education	93	26.3
Primary	101	28.5
Secondary and above	160	45.2

### Behavioral and health status information of patients

Two hundred twenty-five (63.6%) of the ART patients drank alcohol, while 38 (10.7%) used tobacco and chatted. Almost all of the patients, 334 (94.4%), disclosed their HIV infectivity. Three hundred six (86.4%) of the patients in the study were working. Three hundred forty (96.1%) of the total patients in the study were found to be adhering to their ART well. Adherence is defined as taking ART medications exactly as prescribed. It is also necessary to take them at the appropriate time. It also includes adhering to any special dietary restrictions and abstaining from substance use. Three hundred thirty-two (93.8%) of patients had not been infected with tuberculosis (Table 2).

**Table 2:** Summarized information on HIV/AIDS patients’ clinical and risk behavior factors under ART (University of Gondar teaching hospital, 2009–2015)

Variables	Frequency	Percent
Alcohol consumption		
No	225	63.6
Yes	129	36.4
Tobacco and chat use		
No	316	89.3
Yes	38	10.7
HIV disclosure status		
Not disclosed	20	5.6
Disclosed	334	94.4
Functional status		
Bedridden	8	2.3
Ambulatory	40	11.3
Working	306	86.4
ART adherence level		
Poor	10	2.8
Fair	4	1.1
Good	340	96.1
History of TB disease		
No	332	93.8
Yes	22	6.2

### Description of continuous covariates/measures

The average age of patients receiving ART was 34.8 years (± 9.5 SD). The patients’ average baseline weight was 51.2 kg (± 9.8 SD), and their average current weight was around 54.8 kg (±10.6 SD). The average CD4 cell count at baseline was 183.6 cells/l (±118.6). The mean baseline lymphocyte count was 33.1 cells/mm3 (±19.4) and the baseline hemoglobin was 13.2 g/dl (±3.61894), respectively (Table 3).

**Table 3:** Description of continuous covariates/measures of 354 HIV/AIDS patients under the study (University of Gondar teaching hospital, 2009–2015)

Variables	Measures			
	Minimum	Maximum	Mean	Std. Deviation
Age	16.00	70.00	34.7740	9.54665
Baseline weight	15.00	80.00	51.2062	9.81103
Current weight	28.00	92.00	54.7994	10.57320
Baseline CD4 cell count	11.30	1022.00	183.5800	118.58572
Baseline lymphocyte count	1.10	317.00	33.1274	19.42361
Baseline hemoglobin	5.00	36.40	13.2435	3.61894

### Survival time comparison

There were 19 female deaths and two male deaths. The average survival time for urban and rural patients was 104.9 months and 82.3 months, respectively. Patients with one to two family members had the longest mean survival time compared with three to four and more than five family members. Patients who used tobacco and chat had a median survival time of 73.8 months (Table 4).

**Table 4:** Survival time by potential demographic, clinical, and risk variable for HIV/AIDS patients treated under ART (University of Gondar teaching hospital, 2009–2015)

Variable	Number of patients	Number of deaths	Mean survival time	Median survival time	*p*-value
Sex					
Female	211	19	99.893	-	0.004
Male	143	2	104.548	-	
Residence					
Urban	306	19	104.856	-	0.896
Rural	48	2	82.279	-	
Marital status					
Single	76	11	86.732	-	< 0.01
Married	162	4	101.391	-	
Separated/divorced	90	2	105.998	-	
Widowed	26	4	86.761	-	
Religion					
Orthodox	320	18	-	-	0.592
Muslim	29	3	-	-	
Other	5	0	-	-	
Family size					
1–2	201	8	108.070	-	0.001
3–4	91	3	100.170	-	
≥5	62	10	82.682	-	
Educational status					
No formal education	93	13	94.794	-	0.019
Primary	101	5	96.924	-	
Secondary and above	160	3	106.474	-	
Alcohol consumption					
No	225	9	107.231	-	0.018
Yes	129	12	92.048	-	
Tobacco and chat use					
No	316	14	106.865	-	< 0.01
Yes	38	7	62.160	73.80	
HIV disclosure status					
Not disclosed	20	3	85.811	-	0.028
Disclosed	334	18	104.747	-	
Functional status					
Bedridden	8	1	66.847	-	0.008
Ambulatory	40	6	67.033	73.800	
Working	306	14	106.592	-	
ART adherence level					
Poor	10	2	-	-	0.147
Fair	4	0	-	-	
Good	340	19	-	-	
History of TB disease					
No	332	16	105.650	-	< 0.01
Yes	22	5	52.348	-	

The log rank test was used to compare survival functions among covariate subgroups. The survival distributions of the variables sex, marital status, family size, educational status, alcohol consumption, tobacco and chat use, HIV disclosure status, functional status, and history of TB disease differed (*p*-value 0.05) (Table 4). However, there was no difference in survival distribution between the levels on each of the covariates, such as residence, religion, and ART adherence level (*p*-value > 0.05).

**Fig. 1:**
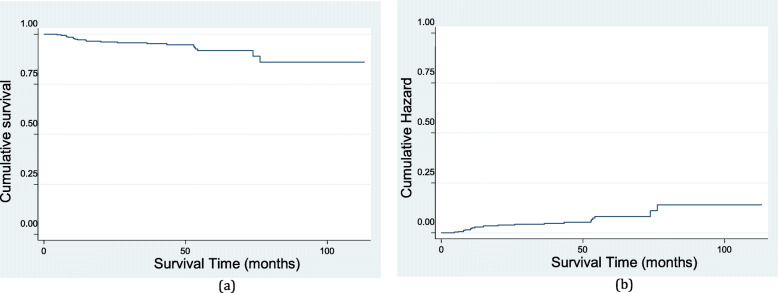
Estimated survival function

Figure 1 a and b show the survival and hazard functions of HIV/AIDS patients who were followed up on ART. Patients who had been followed for more than 75 months had a 0.85 chance of survival (Fig. 1a). The risk of death increased as the number of survival months increased (Fig. 1b). Patients had a constant risk of death after 75 months of follow-up (0.15).

**Fig. 2:**
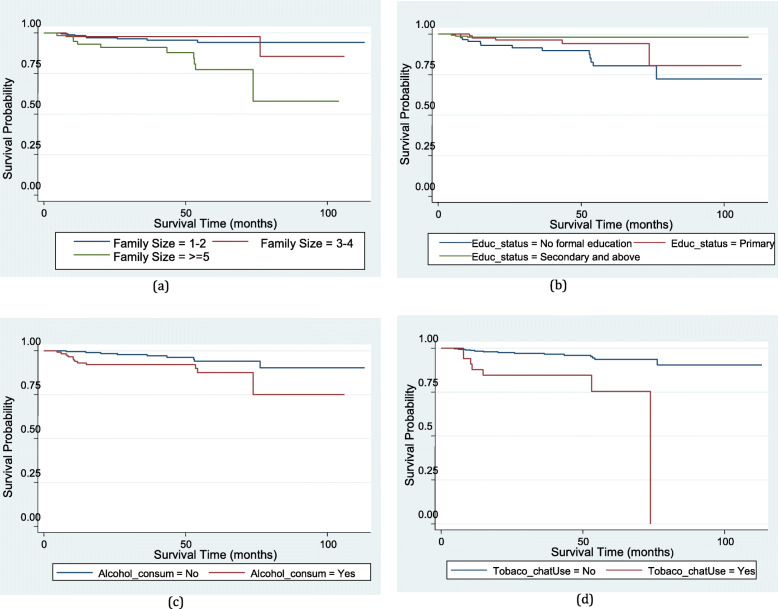
Comparison of survival curves

Figure 2 compares the survival curves of different subgroups/levels of covariates of HIV/AIDS patients on ART. According to the Kaplan-Meier survival function, patients with five or more family members had a lower survival experience than patients with fewer family members as follow-up time increased (Fig. 2a). HIV patients’ survival rates were determined by their educational level. Patients with secondary and higher educational levels had a slightly better chance of survival for longer survival times (Fig. 2b). The Kaplan-Meier survival plot also revealed that patients who drank alcohol, chew chat, and smoked cigarettes had a lower survival probability as survival time increased (Fig. 2c, d).

### The hazard of death in HIV/AIDS patients

Table 5 summarizes all covariates that had a significant association with HIV/AIDS patients’ survival time and risk of death. Age, family size, educational status, alcohol consumption, tobacco and chat usage, baseline weight, current weight, baseline CD4 cell count, baseline lymphocyte count, baseline hemoglobin, and history of TB disease were all associated with HIV patient survival time under ART follow-up (*p*-value 0.05).

**Table 5:** The hazard estimates of covariates associated with the survival time of HIV/AIDS patients under ART follow-up (University of Gondar teaching hospital, 2009–2015)

Predictor variables	***B***	SE	Wald	df	Sig.	AHR	95.0% CI for HR	
							Lower	Upper
Age	.049	.024	4.228	1	.040*	1.051	1.002	1.101
Family size								
≥ 5 (ref)			9.854	2	.007*			
1–2	−1.791	.604	8.785	1	.003*	.167	.051	.545
3–4	−2.120	.852	6.197	1	.013*	.120	.023	.637
Educational status								
Secondary and above (ref)			6.612	2	.037*			
No formal Education	1.616	.762	4.491	1	.034*	5.032	1.129	22.423
Primary	.213	.858	.061	1	.804	1.237	.230	6.646
Alcohol consumption								
Yes (ref)								
No	−1.225	.623	3.873	1	.049*	.294	.087	.995
Tobacco and chat use								
Yes (ref)								
No	−2.296	.646	12.633	1	.000*	.101	.028	.357
Baseline weight	−.084	.042	4.019	1	.045*	.920	.847	.998
Current weight	−.074	.034	4.900	1	.027*	.928	.869	.992
Baseline CD4 cell count	−.010	.003	7.895	1	.005*	.990	.984	.997
Baseline lymphocyte count	−.035	.023	2.296	1	.130	.966	.924	1.010
Baseline hemoglobin	−.224	.066	11.400	1	.001*	.800	.702	.910
History of TB disease								
Yes (ref)								
No	−1.932	.703	7.552	1	.006*	.145	.037	.575

*df* Degree of freedom, *ref* Reference category, *AHR* Adjusted hazard ratio

*Significant (*p*-value < 0.05)

The estimated hazard ratio in Table 5 can be used to interpret the effects of each covariate. Age had a significant effect on HIV patient survival time (HR =1.051, 95% CI 1.002, 1.101). The risk of death for HIV/AIDS patients increased by 5.1% per year.

Another significant predictor of patient survival time was family size. The risk of death for HIV/AIDS patients on ART with one to two family members was 0.165 times lower than for patients with five or more family members (HR = 0.165, 95% CI 0.051, 0.545). Patients with three to four families had an 88% lower risk of death than those with five or more patients (HR = 0.120, 95% CI 0.023, 0.637).

The risk of death for illiterate HIV/AIDS patients on ART follow-up was 5.032 times higher than for patients with a secondary or higher educational status (HR = 5.032, 95% CI 1.129, 22.423).

HIV/AIDS patients who did not consume alcohol were 0.294 times less likely to die than those who did (HR = 0.294, 95% CI 0.087, 0.995). The risk of death for patients who did not use tobacco or chat was 89.1% lower than for patients who did (HR = 0.101, 95% CI 0.028, 0.357).

The baseline weight of HIV patients was also linked to their survival time. As a patient’s baseline weight increased by 1 kg, the risk of death decreased by 8% (HR = 0.920, 95% CI 0.847, 0.998). A patient’s risk of death was also reduced by 7.2% for every kilogram increase in current weight (HR = 0.928, 95% CI 0.869, 0.992).

A one-cell/μl increase in baseline CD4 cell count can reduce a patient’s risk by 1% (HR = 0.990, 95% CI 0.984, 0.997). As baseline hemoglobin increased by 1 g/dl, the risk of death in an HIV patient decreased by 20% (HR = 0.800, 95% CI 0.702, 0.910).

HIV patients with a negative TB history had an 85.5% lower risk of death than those with a positive TB history (HR = 0.145, 95% CI 0.037, 0.575).

## Discussion

The classical techniques were used in this study to analyze risk factors for the survival time of HIV/AIDS patients on ART follow-up. Using the Cox proportional hazards model, a number of variables were used to explain the variation in HIV patient survival time. According to the findings of this study, 5.91% of the patients died. In comparison to other similar studies [6, 8–10], this death rate was very low. The reason for the low reported figure could be due to the higher level of adherence to ART among patients in the study area.

The age of the patient was found to be a significant predictor of HIV patient survival time. Patients who were older had a lower chance of survival than those who were younger. This result is also consistent with previous research in Ethiopia [6, 9] and Brazil by [10]. This could be because the immune recovery of older people has slowed.

The gender association with survival experience was found to be non-significant in this study. Previous research [5, 11, 12] discovered the same result. The number of family members within the patient contributed to the patient’s survival time. In this study, we discovered that patients with one to two families survived for a longer period of time than patients with large families (specifically, five or more). In a limited economy, it may be difficult to provide a balanced and timely diet for the patient if there are more dependents and non-fertile elderly people in the household.

Educational attainment was also a statistically significant predictor of patient survival. Patients who were illiterate had a lower survival rate than those who had a primary or higher education level. This result was consistent with the findings of other research [10, 13]. This figure is most likely the result of less educated patients’ lack of psychological, mental health care, and economic preparedness.

Other influential covariates for patient survival include alcohol consumption, chat, and tobacco use. Drinking alcohol, smoking, and using chat, as well as using other drugs, can all have a negative impact on a patient’s immune system and hasten the progression of the disease. Drinking or using drugs can also have an impact on a patient’s adherence to HIV treatment.

The baseline and current weights had a statistically significant effect on AIDS patients’ chances of survival. This findings is consistent with those of other studies [6, 9]. The effect of weight gain on HIV mortality can be justified that weight gain may be associated with good nutrition and higher BMI. Studies in people living with HIV infection have demonstrated that higher BMI is associated with higher CD4^+^ cell count, lower HIV viral load, reduced risk of opportunistic infections, slower progression to AIDS, and reduced mortality [14–21], and that weight loss is associated with accelerated disease progression contributing to increased mortality [22, 23].

In this study, we discovered that patients’ survival time was determined by their baseline CD4 cell count. Patients with a lower CD4 cell count had a lower chance of survival. This study was conducted at the same time as two other similar studies [10, 13]. The CD4+ count is an important biological marker of immune status [24–26] and an indicator of late diagnosis and treatment. Current international consensus recommendations for starting treatment are based on CD4+ counts, viral loads, and clinical data [27, 28].

This study found that survival time was statistically associated with baseline hemoglobin. The study results also indicated an association between positive TB status (patients with TB) and lower survival, which corroborates the finding of another study [13].

## Conclusion

This study found that there were far fewer deaths than in previous studies of a similar nature. Age, family size, educational status, alcohol consumption, tobacco and chat usage, baseline weight, current weight, baseline CD4 cell count, baseline lymphocyte count, baseline hemoglobin, and TB history were found to be significantly associated with the survival experience of HIV patients on ART fellow-up. Patients must abstain from alcohol in order to improve their chances of survival, and they must eat balanced diets in order to avoid weight loss. Furthermore, healthcare workers must provide patients with health information about risk factors for poor survival.

## Data Availability

Data will be made available upon requesting the primary author.

## Abbreviations

μl: Microliter
AHR: Adjusted hazard ratio
AIDS: Acquired immune deficiency syndrome
ART: Antiretroviral therapy
BMI: Body mass index
CD4: Cluster of differentiation 4
CI: Confidence interval
df: Degree of freedom
g/dl: Gram per deciliter
HIV: Human immunodeficiency virus
HR: Hazard ratio
kg: Kilogram
mm^3^: Cubic millimeter
SD: Standard deviation
SPSS: Statistical package for social sciences
TB: Tuberculosis
